# Dendritic Cells Expressing MyD88 Molecule Are Necessary and Sufficient for CpG-Mediated Inhibition of IgE Production In Vivo

**DOI:** 10.3390/cells8101165

**Published:** 2019-09-28

**Authors:** Ricardo W. Alberca Custodio, Luciana Mirotti, Eliane Gomes, Fernanda P.B. Nunes, Raquel S. Vieira, Luís Graça, Rafael R. Almeida, Niels O. S. Câmara, Momtchilo Russo

**Affiliations:** 1 Institute of Biomedical Sciences, Department of Immunology, University of Sao Paulo, Sao Paulo 05508-000, Brazil; Ricardowesley@gmail.com (R.W.A.C.); lmirotti@gmail.com (L.M.); melloeag@gmail.com (E.G.); raqueldesouzavieira@outlook.com (R.S.V.); rafaelbio13@yahoo.com.br (R.R.A.); niels@icb.usp.br (N.O.S.C.); 2 Institute of Molecular Medicine, University of Lisbon, 1649-028 Lisbon, Portugal; lgraca@medicina.ulisboa.pt

**Keywords:** allergy, IgE, IgG2c, anaphylaxis, dendritic cells

## Abstract

Elevated levels of immunoglobulin E (IgE) are associated with allergies and other immunological disorders. Sensitization with alum adjuvant favours IgE production while CpG-ODN adjuvant, a synthetic toll-like receptor 9 (TLR9) agonist, inhibits it. The cellular mechanisms underlying in vivo TLR regulation of immunoglobulin production, specially IgE, are still controversial. Specifically, TLR-mediated IgE regulation in vivo is not yet known. In this study we showed that augmented levels of IgE induced by sensitizations to OVA with or without alum adjuvant or with OVA-pulsed dendritic cells (DCs) were inhibited by co-administration of CpG. Notably, CpG-mediated suppression of IgE production required *MyD88*-expression on DCs but not on B-cells. This finding contrasts with previous in vitro studies reporting regulation of IgE by a direct action of CpG on B cells via MyD88 pathway. In addition, we showed that CpG also inhibited IgE production in a *MyD88*-dependent manner when sensitization was performed with OVA-pulsed DCs. Finally, CpG signalling through MyD88 pathway was also necessary and sufficient to prevent anaphylactic antibody production involved in active cutaneous anaphylaxis.

## 1. Introduction

Over the past five decades the prevalence and incidence of allergic disease have increased worldwide. Atopy is a term used to describe a group of diseases such as allergic rhinitis, hay fever, asthma, atopic eczema and food allergy in individuals that develop an immediate immunoglobulin E (IgE)-mediated hypersensitivity to otherwise harmless environmental antigens [1]. The work of Shirakawa et al. showing a strong inverse association between serum levels of IgE and delayed type hypersensitivity to Mycobacterium tuberculosis antigens among Japanese schoolchildren [2] lend support to the “hygiene hypothesis” that postulated an inverse relationship between allergy and infections [3]. Clinical studies indicate that murine and human IgE share many characteristics in their regulation and production [4]. Classically, IgE and IgG1 antibody switching is mediated by interleukin-4 (IL-4)-producing T helper 2 (Th2) cells while interferon gamma (IFNγ)-producing Th1 cells favour IgG2a switching [5]. Therefore, it is expected that Toll-like receptors (TLRs) agonists that are viewed as Th1 adjuvants would induce Th1-associated isotypes while alum, that is considered a Th2 adjuvant, would favour IgE production [6]. However, despite its Th2-promoting activities, alum has been used empirically in many anti-microbial vaccines formulations [7]. We have previously shown that adsorption of lipopolysaccharide (LPS), a TLR4 agonist, to alum-based tetanus toxoid vaccine dampens toxoid-induced IgE production but enhances IgG1 and IgG2a antibody production [8]. More recently, using a similar approach of absorbing TLRs agonists to alum, we found the TLR9 agonist composed of oligodeoxynucleotides (ODN) containing CpG motifs (hereafter referred to as CpG) was the most effective among the TLRs agonists in dampening IgE production [9]. Since CpG is another TLR adjuvant approved for use in humans [10] we were interested in dissecting the mechanism(s) by which alum-based CpG formulation regulates immunoglobulin class switching. Moreover, studies to ascertain the molecular mechanisms governing IgE production in vivo and its regulation by adjuvants have not been addressed. Previous studies performed in vitro have indicated that CpG-regulates IgE and IgG1 production acting directly on B-cells expressing MyD88 molecule [11]. Here we used an established OVA-model of respiratory allergy that induces high levels of total and OVA-specific IgE and low levels of IgG2c antibodies and focused on the role of MyD88 molecule expressed on CD19-positive B cells or on CD11c-positive DCs in CpG-mediated regulation of isotype class switching. We show that addition of CpG to OVA/alum suppressed IgE production and increased IgG2c production in mice selectively deleted for MyD88 molecule in B cells but not in DCs indicating, that MyD88-expressing DCs are necessary and sufficient to suppress IgE and to enhance IgG2c production induced by sensitization with CpG-containing adjuvant formulation.

## 2. Materials and Methods

### 2.1. Animals

Six-to-eight-week-old female C57BL/6 mice (WT), MyD88-KO, RAG-KO or Myd88 fl/fl and mice expressing the recombinase Cre under the control of the CD19 or Itgax (CD11c) promoter [12] were originally purchased from Jackson Laboratories (Bar Harbor, ME) and kept in a specific pathogen-free breeding unit at the Institute of Biomedical Sciences of the University of Sao Paulo (ICB IV-USP). CD11cMyD88^−/−^ (DC-MyD88^−/−^) mice and CD19cMyD88^−/−^ (B-MyD88^−/−^) mice with specific deletion of MyD88 adaptor molecule in either CD11c-positive or CD19-positive cells were generated by breeding respectively CD11c-Cre or CD19-Cre with Myd88flox mice. Proper littermates for the DC-MyD88^−/−^ group (CD11cMyD88^+/+^, hereafter referred as DC-MyD88^+/+^) were also generated. Mice from 129 WT strain or mice lacking type I interferon receptor (IFNAR^−/−^) or type II interferon receptor (IFNGR^−/−^) on 129 background were provided by Dr. Luiz Fernando Reis (Ludwig Institute for Cancer Research, São Paulo, Brazil). All mice (three to five animals per cage with a ventilated system; Alesco, Monte Mor, São Paulo, Brazil) were kept under a 12h light/dark cycle and controlled temperature receiving food and water ad libitum and towel paper used for environment enrichment. Mice were treated according to animal welfare guidelines of ICB (Ethic Protocol 009/2015) and to National Ethics Legislation (11.794 Law).

### 2.2. Alum Gel Preparation

The aluminium gel (Al(OH)_3_) was prepared by precipitation of ammonium aluminium sulfate dodecahydrate (AlH_4_(SO_4_)_2_-12 H_2_O, Alfa Aesar, MA, USA) with an excess of 1N NaOH (Synth, Sao Paulo, Brazil). Aluminium hydroxide (Alum) was suspended in water (Milli Q, Ontario, Canada), washed five times and centrifuged at 1000× *g* for 15 min. The final precipitate was suspended again in water and the final concentration was determined by calculating 1 mL of dry solution.

### 2.3. Experimental Protocol

Mice were subcutaneously (s.c.) sensitized with either 4 µg of ovalbumin (OVA) (Sigma-Aldrich, St. Louis, MO, USA) or with OVA plus 10 µg CpG (CpG-ODN 2395 Class C, a TLR9 agonist purchased from Invivogen San Diego, CA, USA) with or without alum adjuvant gel (1.6 mg) on days 0 and 7. Mice were intra-nasally (i.n.) challenged with 10 µg of OVA in 40 µL of PBS on days 14 and 21. Control mice consisted of naïve non-manipulated animals. In some experiments mice were sensitized with bone marrow-derived dendritic cells pulsed with OVA or OVA plus CpG on days 0 and 7 (see methods below). All treatment procedures were done under anaesthesia with Ketamine 10 mg/kg (Rhobifarma, São Paulo, Brazil) plus Xylazine 100mg/kg (Rhobifarma, São Paulo, Brazil) by the intraperitoneal (i.p.) route. Animals were euthanized by inhaled isoflurane (Cristália, São Paulo, Brazil) 24 h after the last challenge and samples were collected. The OVA used throughout the study was diluted in PBS (2 mg/mL) and depleted of the endotoxin (LPS) activity (measured by Limulus amoebocyte lysate QCL-1000 kit from BioWhittaker, Walkersville, MD, USA) using six to eight cycles of Triton X-114 extractions. Endotoxin level of purified OVA (2 mg/mL) was below the limit of detection by the Limulus assay lysate (less than 0.1 Endotoxin Units).

### 2.4. Generation of Bone Marrow Derived Dendritic Cells

Briefly, bone marrow-derived dendritic cells (BM-DCs) were obtained from femurs of C57BL/6 or DC-MyD88^−/−^ mice. Both ends of the femurs were cut after their removal under sterile conditions and flushed with a syringe containing 5 mL of RPMI-1640 medium (Sigma-Aldrich, St. Louis, MO, USA) with 10% foetal bovine serum (FBS) (Sigma-Aldrich, St. Louis, MO, USA). Bone marrow cells were plated into 6-well plates at 1 × 10^6^ cell/well and cultured at 37 °C in 5% CO_2_ from day 0 to 4 with 20 ng/mL of GM-CSF (BioLegend, San Diego, CA, USA) and supplemented with more GM-CSF on day 4 [13]. On day 6, the cells were either primed with OVA (100 µg/well) or OVA plus CpG (1 µg/well). On day 7, BM-DCs were harvested, washed with PBS, counted and 1 × 10^6^ cells were used for the s.c sensitization.

### 2.5. ELISA for Antibody Determinations

Blood samples were collected by cardiac puncture, centrifuged and serum was stored at −20 °C. Serum antibodies were determined by Enzyme-linked immunosorbent assay (ELISA). Total IgE concentrations were determined by sandwich-ELISA using kit BD OptEIA ELISA Set (BD, San Diego, CA, USA). OVA-specific IgE levels were determined by adding serum samples at 1/10 dilutions to plates coated with rat anti-mouse IgE (SouthernBiotech, Birmingham AL, USA). After washing, biotin-labelled OVA was added and reaction revealed using ExtrAvidin (Sigma-Aldrich, St. Louis, MO, USA) plus substrate. OVA-specific IgE serum concentrations were calculated using a Chondrex kit (Chondrex, Redmond, WA, USA) with known concentrations of OVA-specific monoclonal IgE antibody. OVA-specific IgG1 and IgG2c were measured by coating the plates with 20 µg/mL of OVA. Serum samples were added at multiple dilutions and revealed with goat anti-mouse IgG1 or IgG2a conjugated to HRP (SouthernBiotech, Birmingham, AL, USA), which also reacts with IgG2c isotype. Standard OVA-specific curves for IgG1 and IgG2c were purchased from Chondrex. All ELISA were performed in 96-well maxisorp plates (Thermo Fisher Scientific, Rochester, NY, USA).

### 2.6. Active Cutaneous Anaphylaxis (ACA) Assay

Mice were sensitized as described above and challenged intranasally on day 14. On day 20, the dorsal region was shaved with a trimmer ER389 (Panasonic, Osaka, Japan). Twenty-four hours later, the animals received an intradermal injection of either OVA (10 µg) or PBS and an intravenous injection of 100 µL of Evans Blue dye (1 mg/mL) immediately thereafter. Thirty minutes later, the animals were sacrificed and skin of the dorsal region was removed for photographic registration. Skin spots were excised and weighted. Dye extraction from skin samples was performed using formamide (8 mL/mg of dry weight) for 72 h [14] and anaphylaxis was quantified by measuring dye absorbance at 620 nm. Results are expressed as µg/mL determined by a standard curve with known concentrations of Evans blue dye.

### 2.7. Statistical Analysis

Statistical analyses were performed using GraphPad Prism (V.5; GraphPad Software). One-way ANOVA followed by Tukey post-test was used. Differences were considered statistically significant when *p* value ≤ 0.05. Data represent the mean ± SD.

## 3. Results

### 3.1. CpG Regulates Lung Allergic Inflammation and Immunoglobulin Production

**Figure 1:**
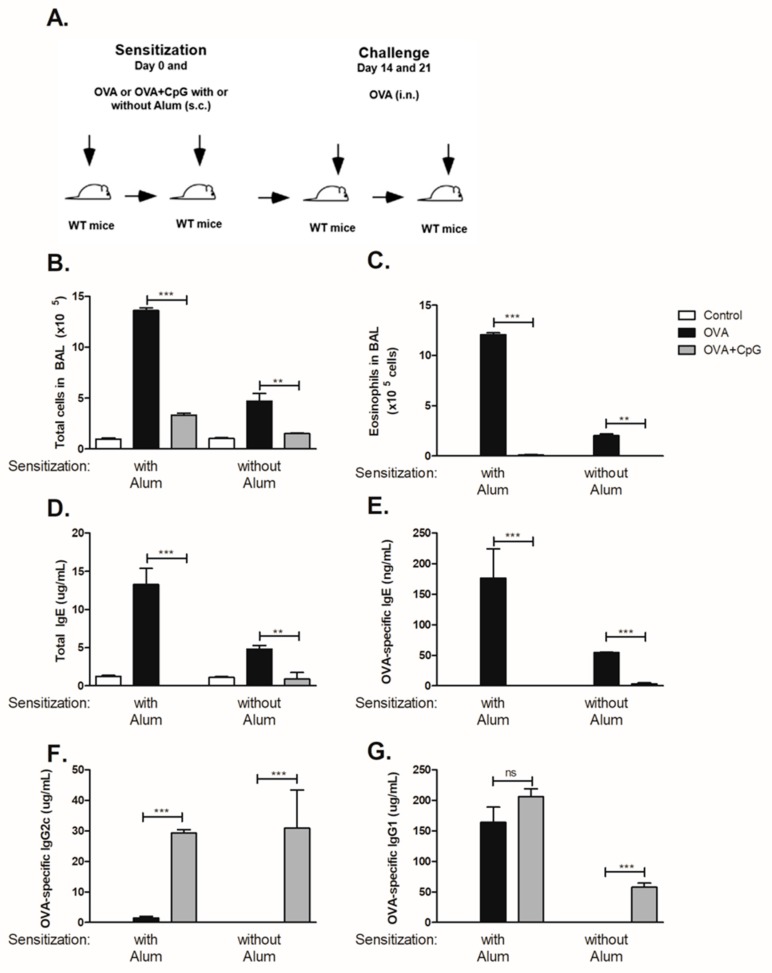
CpG inhibits IgE and enhances IgG production. C57BL/6 wild-type (WT) mice were subcutaneously sensitized with ovalbumin (OVA) or OVA plus CpG (OVA+CpG) with or without alum adjuvant on days 0 and 7 and challenged intranasally with OVA on days 14 and 21. Experiments were performed on day 22. Control mice consisted of non-manipulated animals. (**A**) Schematic experimental protocols. Numbers of (**B**) Total Cells and (**C**) Eosinophils in BAL. Serum levels of (**D**) Total IgE, (**E**) OVA-specific IgE, (**F**) OVA-specific IgG2c and (**G**) OVA-specific IgG1. Groups sensitized with alum adjuvant (*n*  =  5) and without alum (*n*  =  3). Values represent the mean ± SD and are representative of two independent experiments. One-way ANOVA: ** *p*  <  0.01; *** *p*  <  0.001.

To determine the influence of CpG in OVA sensitization and subsequent OVA-induced airway eosinophilic allergic inflammation and OVA-specific antibody production we subcutaneously sensitized C57BL/6 mice to OVA or to OVA + CpG in the presence or absence of alum adjuvant and then challenged the mice with OVA as depicted in Figure 1A. We found that sensitization to OVA induced eosinophilic allergic inflammation that was more intense in the presence of alum as revealed by total cell and eosinophil counts in sensitized mice when compared to non-manipulated control mice (Figure 1B,C). Addition of CpG to OVA during sensitization, in presence or absence of alum decreased allergic inflammation (Figure 1B,C), confirming and extending our previous results [9]. Mice sensitized to OVA with alum and challenged with PBS did not develop allergic lung disease and showed low levels of total IgE (<2 µg/mL) and OVA-specific IgE (<30 ng/mL). In OVA-sensitized and OVA-challenged mice, sensitization to OVA with alum adjuvant resulted in roughly 3-fold higher levels of total and OVA-specific IgE than that obtained in mice sensitized to OVA without alum adjuvant (Figure 1D,E). In contrast, sensitization to OVA in the presence of CpG inhibited IgE production (Figure 1D,E). Sensitization to OVA with alum induced respectively low and high concentrations OVA-specific IgG2c and IgG1 antibodies (Figure 1F,G) whereas in mice sensitized to OVA without alum the concentrations of both OVA-specific IgG2c and IgG1 isotypes were negligible (Figure 1F,G). Notably, sensitization to OVA with alum in the presence of CpG significantly augmented OVA-specific IgG2c but not IgG1 antibody production (Figure 1F,G) whereas in mice sensitized to OVA without alum adjuvant, the production of OVA-specific IgG2c as well as OVA-specific IgG1 significantly increased compared to OVA group (Figure 1F,G). Collectively, we conclude that high levels OVA-specific IgG1 production were obtained when alum adjuvant was used whereas in adjuvant-free conditions specific IgG1 was only induced by addition of CpG. The fact that CpG increased IgG2c and IgG1 levels but decreased IgE production when sensitization was performed without alum adjuvant implies that increased IgG1 production cannot be correlated with increased IgE production. In contrast, when alum was used for sensitization, addition of CpG did not increase IgG1 levels but increased IgG2c production and decreased IgE production. Therefore, IgG1 production is not necessarily associated with increased IgG2c levels or with decreased IgE production. Therefore, in all subsequent experiments we focused mainly on IgE and IgG2c production. All in all, it is clear that CpG negatively regulates IgE production while IgG2c production is positively regulated, regardless of whether alum is used or not for OVA sensitization.

### 3.2. MyD88 Expression on B Cells Is Not Essential for CpG-Mediated Regulation of IgE and IgG2c Isotype Production In Vivo

**Figure 2:**
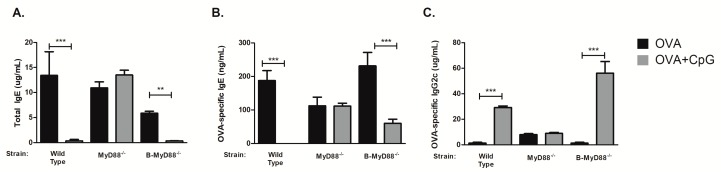
Myd88 expression on B-cells is dispensable for CpG-mediated inhibition of IgE production. C57BL/6 wild-type (WT) or mice lacking Myd88 expression (MyD88^−/−^) in all cells or in B lymphocytes (B-MyD88^−/−^) mice were subcutaneously sensitized with ovalbumin (OVA) or OVA plus CpG using alum as adjuvant on days 0 and 7 and challenged intranasally with OVA on days 14 and 21. Experiments were performed on day 22. Serum levels of (**A**) Total IgE, (**B**) OVA-specific IgE and (**C**) OVA-specific IgG2c. OVA groups (*n* =  5) and OVA+CpG groups (*n* =  5). Values represent the mean ± SD and are representative of two independent experiments. One-way ANOVA: ** *p*  <  0.01; *** *p*  <  0.001.

We first determined the requirement of MyD88 molecule using mice with complete lack of MyD88 molecule since CpG is thought to signal mainly via MyD88 pathway. We found that sensitization to OVA in the presence of CpG did not block IgE production in MyD88^−/−^ mice when compared to WT mice (Figure 2A,B), indicating the essential role of MyD88 molecule in mediating the inhibition of IgE production. In the same vein, CpG did not enhance IgG2c production in MyD88^−/−^ mice (Figure 2C). Concentrations of OVA-specific IgG1 antibodies were similar in both groups (around 200 µg/mL). Results obtained in MyD88-deficient mice indicate that MyD88 molecule is essential for CpG-regulation of isotype class switching. However, the target cell type involved in this regulation remained elusive. It has been shown in vitro that CpG acts directly on purified B cells inhibiting IgE production and enhancing IgG2a production [11]. Importantly, sensitization to flagellin (a protein antigen and TLR5 agonist) adsorbed to alum, resembling our protocol (OVA + CpG/alum) of sensitization, suggested that the generation of T cell-dependent antigen-specific antibody responses required the activation of B cells via MyD88 pathway [15]. Therefore, we next determined the effect of CpG in mice with selective deficiency of *Myd88* gene in B cells using Cre-lox technology to delete *Myd88* gene in CD19-positive cells (B-MyD88^−/−^). We found that CpG inhibited IgE production in mice bearing *Myd88*-deficient B cells (Figure 2A,B) clearly indicating that *Myd88* expression on B cells is dispensable for the inhibition of IgE production. We conclude that *Myd88* expression is fundamental for down regulation of IgE production by CpG but *Myd88* expression on B cells is not necessary for this effect.

### 3.3. CD11c-Positive Dendritic Cells Expressing MyD88 Molecule Are Necessary and Sufficient for CpG-Induced Inhibition of IgE and Augmented IgG2c Production

**Figure 3:**
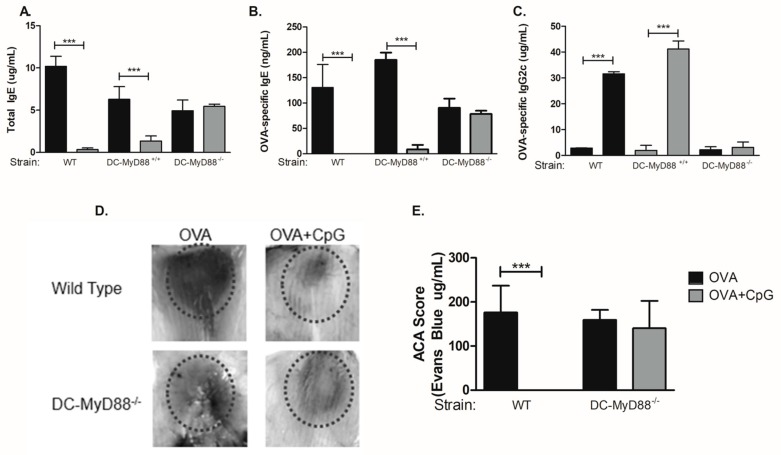
Myd88 expression on CD11c-positive cells mediates CpG-induced regulation of IgE and IgG2c production. C57BL/6 wild-type (WT) or mice lacking total Myd88 expression on CD11c-positive dendritic cells (DC-MyD88^−/−^) or their control littermates (DC-MyD88^+/+^) mice were subcutaneously sensitized with either ovalbumin (OVA) or OVA plus CpG using alum as adjuvant on days 0 and 7 and challenged intranasally with OVA on days 14 and 21. Experiments were performed on day 22. Serum levels of (**A**) Total IgE, (**B**) OVA-specific IgE or (**C**) OVA-specific IgG2c were measured by ELISA. Active cutaneous anaphylaxis (ACA) assay measured by Evans blue dye extravasation upon intradermal OVA injection was determined on day 21. (**D**) Representative skin pictures of Evans blue extravasation and (**E**) ACA score determined by measuring Evans blue extracted from the tissue. OVA groups (*n*  =  5) and OVA+CpG groups (*n*  =  5). Values represent the mean ± SD and are representative of two independent experiments. One-way ANOVA: *** *p*  <  0.001.

Our results indicated that B cells were dispensable for CpG-mediated regulation of IgE and IgG2c isotypes. Therefore, we focused our study on the role of *Myd88* expression on other cell types that respond to CpG signalling and are involved antigen presentation, namely dendritic cells (DCs). For this we used mice with specific deletion of *Myd88* gene on cells expressing CD11c integrin (DC-MyD88^−/−^). As a control group we used WT and littermates mice with MyD88-expressing MyD88 CD11c-positive cells (DC-Myd88^+/+^). Sensitization with OVA plus CpG adsorbed on alum inhibited IgE production in WT and DC-Myd88^+/+^ littermates but not in DC-Myd88^−/−^ mice (Figure 3A,B). Likewise, OVA-specific IgG2c production was increased in WT and DC-MyD88^+/+^ but not in DC-MyD88^−/−^ mice in OVA+CpG group when compared to OVA group (Figure 3C). OVA-specific IgG1 antibodies were not decreased in the OVA+CpG group when compared to OVA group (data not shown). Therefore, we conclude that CpG signalling through MyD88 molecule expressed on CD11c^+^ putative DCs is necessary and sufficient for IgE inhibition and enhancement of IgG2c production in vivo. In order to characterize functionally anaphylactic antibody activity, we determined the magnitude of active cutaneous anaphylaxis (ACA) that was induced in mice upon intradermal OVA administration in WT or DC-MyD88^−/−^ mice sensitized with OVA/alum or OVA+CpG/alum. We found that both WT and DC-MyD88^−/−^ mice sensitized with OVA/alum developed ACA of similar magnitude as revealed by ACA score based on Evans blue dye extravasation (Figure 3D, left panel and E). In contrast, sensitization with OVA+CpG/alum inhibited ACA in WT but not DC-MyD88^−/−^ mice (Figure 3, right panel and E). These results reinforce that CpG inhibits the production of anaphylactic antibodies.

### 3.4. CpG-Induced Inhibition of IgE and Augment IgG2a Production Is Independent of Type I or Type II Interferon Receptors

**Figure 4:**
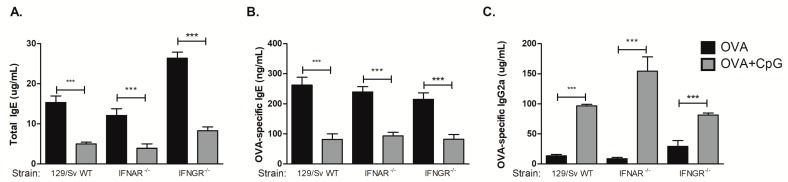
CpG-induced inhibition of IgE and augmented IgG2a production is independent of type I or type II interferon receptors. 129 WT strain or mice lacking type I interferon receptor (IFNAR^−/−^) or type II interferon receptor (IFNGR^−/−^) on 129 background were submitted to the same protocol of OVA sensitization with alum and i.n. OVA challenge. Experiments were performed on day 22. Serum levels of (A) total IgE, (B) OVA-specific IgE or (C) OVA-specific IgG2c were measured by ELISA. OVA groups (*n*  =  5) and OVA+CpG groups (*n*  =  5). Values represent the mean ± SD and are representative of two independent experiments. One-way ANOVA: *** *p*  <  0.001.

We have previously shown that type I cytokines or type I and II interferon (IFN) receptors were not required for CpG-induced inhibition of IgE production, however we had not determined the regulation of IgG isotypes yet [9]. Therefore, we performed experiments using the 129 mouse strain with deficiencies in type I or type II IFN receptors. The 129 strain mice carry the Igh^a^ haplotype and therefore produce IgG2a but not the IgG2c isotype [16]. We found that CpG inhibited IgE production in WT as well as in IFNAR^−/−^ or IFNGR^−/−^ 129 mice (Figure 4A,B), confirming our previous results that type I or type II INF receptors are not involved in CpG-mediated inhibition of IgE [9]. Notably, we found that CpG augmented IgG2a antibody production in these mouse strains (Figure 4C). We conclude that deficiency of type I or type II interferon receptors *per se* did not significantly influence the regulation of isotype class switching by CpG.

### 3.5. MyD88 Expression on OVA-primed Dendritic Cells Is Essential for Inhibition IgE Production by CpG

**Figure 5:**
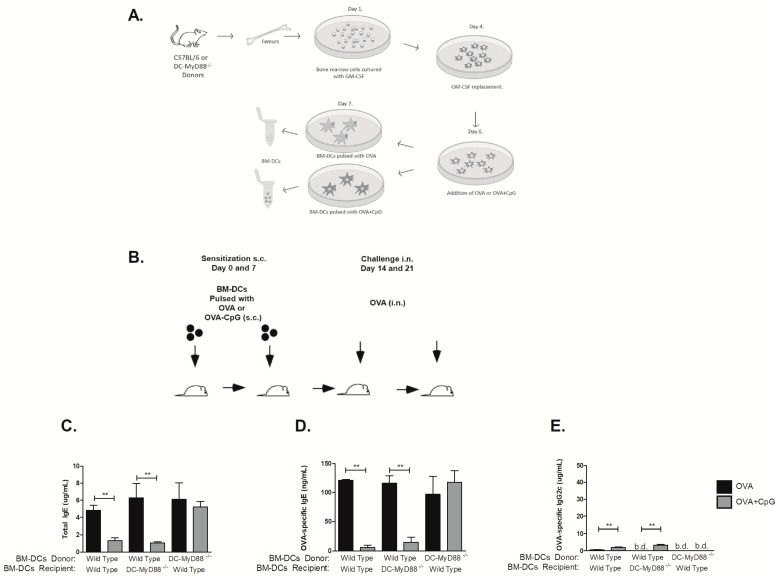
Myd88 expression on CD11c-positive dendritic cells is essential for CpG-induced suppression of IgE production. Bone marrow-derived dendritic cells (BM-DCs) obtained from C57BL/6 wild type (WT) or mice lacking Myd88 on CD11c-positive dendritic cells (DC-MyD88^−/−^) donors were pulsed with OVA or OVA plus CpG and injected subcutaneously on days 0 and 7 respectively to DC-MyD88^−/−^ or WT recipient mice. On days 14 and 21 recipient mice were challenged intranasally with OVA Experiments were performed on day 22. (**A**) Schematic protocol for bone marrow derived dendritic cells (BM-DCs), (**B**) Experimental protocol. Serum levels of (**C**) Total IgE, (D) OVA-specific IgE or (E) OVA-specific IgG2c. OVA groups (*n*  =  5) and OVA+CpG groups (*n*  =  5). Values represent the mean ± SD and are representative of two independent experiments. b.d.—bellow detection level and One-way ANOVA: *** *p*  <  0.001, different from respective OVA group.

Given the crucial role that MyD88 molecule expressed on DCs exerts on CpG-induced immunoglobulin class switching we next determined whether similar regulation could be obtained using purified DCs for sensitization. For this, bone marrow derived dendritic cells (BM-DCs) were differentiated in vitro from either WT or DCs-*Myd88*^−/−^ donor mice and were pulsed with OVA alone or OVA plus CpG as depicted in Figure 5A and injected subcutaneously in WT or DC-*Myd88*^−/−^ recipients respectively using the protocol depicted in Figure 5B. We found that sensitization with donor DCs pulsed with OVA, expressing or not MyD88 molecule, induced an increase in total and OVA-specific IgE antibodies (Figure 5C,D) upon intranasal OVA challenge. Moreover, animals sensitized with WT BM-DCs but not with DC-Myd88^−/−^, pulsed with OVA plus CpG and transferred to WT recipients, inhibited IgE production (Figure 5C,D) indirectly indicating that *MyD88* expression on DCs is fundamental for CpG-induced IgE inhibition. Surprisingly, OVA-specific IgG1 antibody levels were bellow detection in all groups studied (data now shown). Moreover, OVA plus CpG-pulsed DCs obtained from WT but not from DC-Myd88^-/-^ donor mice induced a very low concentration (~5 µg/mL) of OVA-specific IgG2c antibodies (Figure 5C). The results with OVA-pulsed DCs indicate that this type of sensitization is not sufficient to induce significant production of IgG isotypes and reinforce our results indicating that *Myd88* expression on DCs is necessary and sufficient for CpG-mediated inhibition of IgE production.

## 4. Discussion

In the present work we focused on the regulation of immunoglobulin class switching in mice sensitized to OVA in the presence or absence of alum or CpG. We found that independently of whether alum adjuvant was used for OVA sensitization, addition of CpG to OVA resulted in inhibition of total and OVA-specific IgE production and in augmented OVA-specific IgG2a or IgG2c production. Therefore, it is clear that CpG inhibits IgE and enhances IgG2a or IgG2c isotypes production. The effect of CpG on OVA-specific IgG1 production was dependent on whether alum adjuvant was used or not for OVA sensitization. In alum-free OVA sensitization, OVA-specific IgG1 antibodies were almost absent while in sensitization to OVA in the presence of CpG, IgG1 levels were significantly higher than that observed with sensitization to OVA alone. In contrast, when alum was used for OVA sensitization, OVA-specific IgG1 production reached high levels that were not further increased significantly with the addition of CpG. Since OVA-specific IgG1 isotype was significantly increased when sensitization of mice to OVA was done in the presence of CpG, a TLR9 agonist that is considered a pro-Th1 adjuvant, the IgG1 isotype cannot be viewed solely as Th2-associated isotype [5]. In this regard, there is a controversy in the literature as to whether TLR signalling is fundamental or not for enhanced production of IgG isotypes [17,18]. Our results reconcile in part this controversy since sensitization to OVA with alum adjuvant induces high levels OVA-specific IgG1 antibodies and as such its production seems to be TLR-independent, a finding that is in line with the work of Gavin et al. [18] while in alum-free sensitization, substantial levels of OVA-specific IgG1 production only occurred in the presence of CpG indicating the involvement of TLR signalling in IgG1 production, which is in agreement with the work of Pasare and Medzhitov [17]. In addition, our data confirm previous findings showing that sensitization to OVA with alum adjuvant induced high levels of IgG1 that were significantly decreased in alum-free sensitization [19] but CpG never decreased IgG1 production. Regarding IgG2a or IgG2c isotypes, our results clearly showed that irrespective of the usage or not of alum adjuvant, CpG significantly augmented OVA-specific IgG2c reinforcing the notion of the potentiating role of TLR signalling for IgG2a/c production.

It was previously shown that CpG induces murine B cells to proliferate and secrete immunoglobulin in vitro and in vivo [20]. Indeed, B cells express TLRs and respond to TLR agonists by differentiating into antibody-secreting cells [21]. In vitro experiments with purified B cells showed that CpG inhibits IgG1 and IgE switching induced by IL-4 and CD40 signalling [11]. This effect correlated with the expression of T-bet mRNA on purified B cells [11]. Another study showed CpG redirects Ig isotype production by regulating the specificity of class switch recombination directly on B cells in a manner critically dependent on TLR9 and myeloid differentiation molecule 88 (MyD88) expression [22]. However, the requirement of TLR-MyD88 signalling in IgG isotype class switching induced in vivo by sensitizations with different adjuvants indicated that MyD88 pathway is either essential or dispensable for enhanced IgG isotype production [17]. Interestingly, it was shown that for regulation of IgG2c isotype by CpG, that is encoded by IgH-1a haplotype [23], the requirement of *Myd88* expression on B-cells or dendritic cells (DCs) and/or other cell types varies according to the type or physical form of CpG formulation used [24,25,26]. We found that *Myd88* expression on DCs but not on B cells, is fundamental for down regulation of IgE production by CpG. In the same vein, we found that enhancement of IgG2c switching by CpG did not require MyD88 molecule on B cells. Our results obtained in vivo with IgE regulation stand in contrast with reports suggesting direct action of CpG on B cells as shown by experiments performed in vitro where CpG inhibited IgE switching induced by IL-4 and CD40 signalling [11] or with a study showing that CpG redirected Ig isotype production towards IgG isotypes by regulating the specificity of class-switch recombination directly on B cells in a manner critically dependent upon TLR9 and MyD88 expression [22] or with a study that suggested the requirement of direct B-cell stimulation by TLR ligands performed in reconstituted B-cell deficient mice with MyD88-deficient B lymphocytes [15]. Our results clearly indicated that CpG signalling through MyD88 molecule expressed on CD11c-positive putative DCs is necessary and sufficient for IgE inhibition and enhancement of IgG2c production in vivo. Pioneering studies of IgG2a or IgG2c regulation reported that all viral infections introduce a unique bias in the subclass selection process that makes IgG2a the predominant antiviral IgG antibody in the mouse in contrast to IgG1 predominance when soluble proteins are used for sensitization [27]. Recent studies with virus like particles (VLPs) or soluble antigens with TLRs agonists clarified the requirement of *Myd88* expression on B cells or DCs and/or other cells for IgG isotypes antibody production [26,28]. Notably, *Myd88* expression on B cells was critical for the production of high levels of IgG2a, IgG2c or IgG2b isotypes when VLPs containing TLR9 or TLR7 ligand or inactivated virus were used for immunizations [26,28]. In contrast and in line with our findings, it was shown that TLR-MyD88 signalling on DCs but not on B-cells, was required for augmented production of IgG isotypes to a soluble protein either mixed with a soluble TLR9 ligand or chemically conjugated to it [26]. However, the DCs-dependency for augmented switching towards IgG2c isotype was lost when the physical form of CpG was changed to a more aggregated form [26]. These findings indicate that the adjuvant used, the site where sensitization is performed and the physical nature of the antigen determine the role of different cell types expressing *Myd88* on the enhancement of IgG production. In our model, up-regulation of IgG2c production clearly required *Myd88* expression on DCs. Reports regarding the effect of CpG on IgE regulation in vivo and anaphylaxis are lacking. We found that sensitization to OVA with CpG consistently inhibited the production of OVA-specific IgE and/or anaphylactic antibodies by a mechanisms that was dependent on MyD88 expression on CD11c-positive putative DCs. Presently, we confirmed at single cell level by FACS analysis that CpG decreases drastically the frequency of B cells-producing IgE (manuscript in preparation). Another intriguing finding was the fact that that CpG regulation of IgE or IgG2a/c isotype switching was independent of type I or type II interferon receptors. Our findings with IgE regulation corroborate previous work showing that type 1 cytokines are dispensable for the suppression of Th2-like immune responses by CpG [29]. In addition our data support the notion that IgG2a antibody production might proceed independently of type I IFN or of type II interferons as previously reported [30]. We speculate that regulation of class-switching by CpG might be mediated by the redundant effect of different types of inflammatory cytokines or by lipid inflammatory mediators [31]. Alternatively, CpG stimulation could mimic the exposure to certain infectious agents, an environmental factor widely suggested to influence allergic processes (hygiene hypothesis) through epigenetic alterations [32]. Therefore, one can speculate that epigenetic factors may also be involved in the outcomes we observed in the regulation of IgE production and allergy. Epigenetic mechanisms such as DNA methylation and histone modifications might play a prominent role in the differentiations of Th cells. For instance, *IL4* and *IL13* loci are hypomethylated in Th2 cells while DNA methylation of *IFNG* is preserved [33]. We envisage that during the process of antigen presentation by DCs to Th cells, concomitant signalling thorough MyD88 pathway preclude the development of a T follicular helper cell subset that drives anaphylactic IgE [34]. The results obtained with sensitization to OVA with OVA-pulsed BM-DCs confirmed our results showing that *Myd88* expression on DCs is necessary and sufficient for CpG-mediated inhibition of IgE production. However, the CpG regulation of IgG1 or IgG2c isotypes in sensitization with OVA-pulsed DCs appears to be a much more complex process since we could not detect substantial production of IgG1 or IgG2c antibodies with OVA-primed DCs. Since IgG1 production was negligible in mice sensitized with OVA-primed DCs we suggest that induction IgE antibodies with OVA-pulsed DCs did not result from sequential switching from IgG1-positive B cells and as such the produced IgE antibodies probably did not suffer affinity maturation [35].

All in all, we demonstrate that DCs are the target cells governing in vivo regulation of IgE and IgG2c antibodies induced by sensitization to OVA with alum-based CpG formulation (see graphical abstract). Our findings might pave the way for the rational use of anti-allergic or anti-microbial vaccine formulations.
